# Neurophysiological Effects of Sleep Deprivation in Healthy Adults, a Pilot Study

**DOI:** 10.1371/journal.pone.0116906

**Published:** 2015-01-21

**Authors:** Ursula M. H. Klumpers, Dick J. Veltman, Marie-Jose van Tol, Reina W. Kloet, Ronald Boellaard, Adriaan A. Lammertsma, Witte J. G. Hoogendijk

**Affiliations:** 1 Department of Psychiatry, VU University Medical Center, Amsterdam, The Netherlands; 2 Department of Nuclear Medicine & PET Research, VU University Medical Center, Amsterdam, The Netherlands; 3 Neuroscience Campus Amsterdam, VU University Medical Center, Amsterdam, The Netherlands; 4 Neuroimaging Center University Medical Center, Groningen, The Netherlands; University of Pennsylvania, UNITED STATES

## Abstract

Total sleep deprivation (TSD) may induce fatigue, neurocognitive slowing and mood changes, which are partly compensated by stress regulating brain systems, resulting in altered dopamine and cortisol levels in order to stay awake if needed. These systems, however, have never been studied in concert. At baseline, after a regular night of sleep, and the next morning after TSD, 12 healthy subjects performed a semantic affective classification functional magnetic resonance imaging (fMRI) task, followed by a [^11^C]raclopride positron emission tomography (PET) scan. Saliva cortisol levels were acquired at 7 time points during both days. Affective symptoms were measured using Beck Depression Inventory (BDI), Spielberger State Trait Anxiety Index (STAI) and visual analogue scales. After TSD, perceived energy levels, concentration, and speed of thought decreased significantly, whereas mood did not. During fMRI, response speed decreased for neutral words and positive targets, and accuracy decreased trendwise for neutral words and for positive targets with a negative distracter. Following TSD, processing of positive words was associated with increased left dorsolateral prefrontal activation. Processing of emotional words in general was associated with increased insular activity, whereas contrasting positive vs. negative words showed subthreshold increased activation in the (para)hippocampal area. Cortisol secretion was significantly lower after TSD. Decreased voxel-by-voxel [^11^C]raclopride binding potential (BP_ND_) was observed in left caudate. TSD induces widespread cognitive, neurophysiologic and endocrine changes in healthy adults, characterized by reduced cognitive functioning, despite increased regional brain activity. The blunted HPA-axis response together with altered [^11^C]raclopride binding in the basal ganglia indicate that sustained wakefulness requires involvement of additional adaptive biological systems.

## Introduction

Lack of sleep is a common condition in everyday life, either related to psychosocial demands or related to working shift hours. In healthy individuals, this may induce decreased alertness and vigilance, together with a general decline in mood. Total sleep deprivation (TSD) has been associated with general psychomotor slowing and diminished cognitive performance [1,2]. In affective disorders, only one night of sleep deprivation may improve mood in 40–60% of subjects with major depressive disorder [3], whereas bipolar patients may even turn into (hypo)mania [4]. Thus, in humans, sleep deprivation is clearly related to altered emotional and affective functioning.

From an evolutionary perspective, staying awake has served to guard against outside threats, requiring increased alertness. Motivational control over the waking state is necessary and presumed to be modulated by top-down cortical control systems, involving prefrontal executive regions [5]. Using [^18^F]-2-fluoro-2-deoxy-D-glucose ([^18^F]FDG) as a ligand in positron emission tomography (PET) studies, sleep deprivation has been associated with reduced metabolic activity in a network of brain regions, including prefrontal and limbic regions, the thalamo-basal ganglia circuit, and cerebellum [6,7]. Neurophysiologically, dopamine (DA) release is supposed to increase wakefulness, partly through the D2 receptor [8,9,10] and partly by acting as a stimulator of corticotropin releasing hormone (CRH) [11]. Ultimately, CRH releases cortisol from the adrenal cortex via the hypothalamic pituitary adrenal (HPA) axis, a key endocrine response mechanism to a stressful situation. These effects are superimposed upon the circadian rhythm of the HPA axis, and largely controlled by the central body clock, the suprachiasmatic nucleus (SCN). HPA axis functioning can be assessed by the cortisol awakening response (CAR), reflecting the natural HPA response to stress of sleep-wake transitions [12]. It is unknown, however, how cortical, dopaminergic and HPA axis activities interact to maintain wakefulness. Studying their interaction may also provide insight into the pathophysiology of depressive disorder, with its frequently occurring sleeping problems and HPA-axis hyperactivity [13,14].

The purpose of this pilot study was to assess how the healthy brain responds to TSD and how compensatory and regulatory stress mechanisms may interact as opposed to future clinical studies in mood disorder. It was hypothesized that wakefulness would be associated with an increase in dopamine release and CRH activation, in the presence of altered emotional functioning.

## Materials and Methods

### Participants

Twelve healthy adults (6 female, mean age 29.2 ± 10.2 years; 6 male, mean age 28.5 ± 4.8 years) were recruited through newspaper advertisements. Exclusion criteria included a lifetime history of psychiatric disorders, as assessed by Mini international neuropsychiatric interview [15] and reported contacts with mental health counselors, previous use of psychotropic medication known to interfere with the dopaminergic system, 1^st^ degree relatives with psychiatric disorder, somatic disorders, pregnancy, use of sleep medication and past or current abuse of psychoactive drugs. All subjects were good sleepers, defined as feeling rested after a night’s sleep, and in good physical health as assessed by medical history, physical examination and routine laboratory tests. On the night preceding TSD, subjects slept 6.6 ± 1.1 hours. Mean body mass index was 21.0 ± 1.4 kg·m^−2^, 2 were cigarette smokers (10 per day), and 10 consumed alcohol (1.5 ± 1.1 units day).

### Ethics Statement

Written informed consent was obtained from all participants. The study protocol was approved by the Medical Ethics Review Committee of the VU University Medical Center in Amsterdam.

### Design and Procedure

Cortisol saliva was collected on both days. At baseline (day 1), after a regular night of sleep at home, all subjects underwent functional magnetic resonance imaging (fMRI) scanning in the morning, followed by a 60min [^11^C]raclopride PET scan. After this scanning session, participants returned to their daily activities, including study and/or work. They returned to the hospital at 22.00h for effectuation of total sleep deprivation. During the night, subjects were monitored by a trained observer and engaged in reading, conversation, short walks on the ward, and board games in a well-lit room. At arrival, urine toxicology was screened and found negative for a subset of dopaminergic and wake enhancing drugs, including cocaine, tetrahydrocannabinol (THC) and amphetamines. Use of alcohol, caffeinated beverages and smoking was prohibited during the night, as on both days in-between scan experiments. At day 2, a light meal was served at 6.00h. After having been awake for about 25 hours, fMRI scanning was repeated, followed by a second [^11^C]raclopride PET scan for all participants. After finishing the scan sessions, subjects were asked to stay awake during the remainder of the day, and to postpone sleep until the evening.

### Psychometric Data

Depressive symptoms over the prior week were assessed using the Beck Depression Inventory [16]. At baseline and before scanning, trait and state anxiety were measured using the Spielberger State-Trait Anxiety Inventory (STAI) [17]. During sleep deprivation, self and observer based visual analogue scales (VAS) were registered every 3 hours, starting at 24.00h and finishing at 12.00h, documenting mood, interest, motor inhibition, speed of thought, self appreciation, energy level and concentration on a scale from 0–100. Psychometric data were analyzed using Statistical Package for the Social Sciences (SPSS) version 15.0 for Windows (SPPS Inc, Chicago, Illinois, USA), using Repeated Measures ANOVA.

### Cortisol Measurements

#### Data acquisition

At the baseline interview, participants were instructed to collect saliva samples using Salivettes (Starstedt, Germany)[18,19], at 7 time points per day. One hour cortisol awakening response (CAR) measurements included three sampling points, immediately after awakening (T1), at +30min (T2) and at +60min (T3). Additional saliva samples were taken at +90min (T4) after awakening, at 14.00h (T5), 17.00h (T6) and 23.00h (T7). Subjects were instructed to write down the exact sampling time. On the following day, samples were collected at identical time points (T8–T14). Eating, smoking, drinking tea or coffee or brushing their teeth was prohibited within 15min before sampling. No dental work was allowed within 24 hours prior to sampling. Samples were stored in a refrigerator and returned by the participant or by regular mail. Salivettes were centrifuged at 2000g for 10min, aliquoted and stored at −80°C. Free cortisol analysis was performed by competitive electrochemiluminescence immunoassay (Architect, Abbott Laboratories, Illinois, USA) [20]. The lower limit of quantification was 2.0nmol·L^−1^, the intra- and inter-assay variability coefficients were less than 9 and 11%.

#### Data analysis

The CAR area under the curve (AUC), with respect to increase (AUC_I_) and to ground (AUC_G_), was calculated. AUC_I_ is calculated with reference to the baseline measurement at T1, ignoring the distance from zero for all measurements, and emphasizing change over time. AUC_G_ is the total area under the curve of all measurements [21]. The mean increase in the 1^st^ hour (MnInc) was calculated by subtracting the baseline value at T1 from the mean of the subsequent values at T2 and T3. Using the real sampling time at T2, T3, T9 and T10, cortisol levels were interpolated using piecewise linear spline to +30 and +60min, in order to derive the individual CAR AUC for identical time points on both days [22]. For AUC_G_ T1-T7 and T8-T14, mixed model analysis was used to include time points available, with missing values being interpolated [23].

### fMRI

#### Task design

We used a semantic emotional classification task adapted from Murphy [24] and Elliot [25], where subjects had to respond as quickly as possible to affective target stimuli and ignore distracter stimuli. The fMRI study consisted of two task sessions (runs), one to be executed at baseline and one after sleep deprivation. Each participant therefore performed two versions of the task, their order randomized across subjects. Each task comprised a blocked design with 16 blocks, programmed in E-prime software (Psychology Software Tools, Inc., Pittsburgh, PA, USA). The first two blocks were practice blocks while being in the magnet, to become acquainted with the task and to reduce anticipation anxiety. Within each session, eight different task conditions were presented twice in a pseudo-randomized order, to generate 16 blocks (Table 1). In each block, 22 trials were presented in a randomized order, half of these being targets, and the other half consisting of distracters. Targets and distracters were defined on the basis of emotional valence, with happy (positive (P)), sad (negative (N)), or neutral (O) words as targets, presented with one of the other categories as distracters (e.g. positive targets with negative distracters). All the words were selected from the Centre for Lexical Information (Celex) Database [26], and matched for frequency of written use and word length. Affective words were selected on high emotional impact (positive words 6.0 ± 1.6 letters, intensity 2.2 ± 0.5; negative words 5.7 ± 0.4 letters, intensity 5.9 ± 1). A baseline neutral condition was included, where targets and distracters were defined on the basis of physical properties (italic (I) vs. regular (R) font), providing similar visual input. Each of the 16 blocks started with a written instruction for a fixed 5s, followed by a 1s rest, in which subjects were instructed to respond as fast as possible to the appropriate task condition by pressing a button with the preferred index finger. Following a fixation cross for 800ms, a word was shown for 500ms to which subjects were allowed to respond within an additional fixed inter-stimulus interval of 900ms. After pressing, the word was no longer visible. At the end of a block, a 1s rest was included prior to the next block.

**Table 1:** fMRI task conditions.

**Condition**	**Target**	**Distracter**	Regressor order*
Neutral word (baseline)	Italic (I)	Regular (R)	1
Neutral word (baseline)	Regular (R)	Italic (I)	1
Positive word	Positive (P)	Neutral (O)	2
Negative word	Negative (N)	Neutral (O)	3
Positive word	Neutral (O)	Positive (P)	4
Negative word	Neutral (O)	Negative (N)	5
Both emotions	Positive (P)	Negative (N)	6
Both emotions	Negative (N)	Positive (P)	7
Task Instructions			8

* Order as used in matrix for computing contrast images

#### Data acquisition

T1-weighted MRI scans were acquired using a 1.5T Sonata MR system (Siemens Medical Solutions, Erlangen, Germany) to exclude anatomical abnormalities and for PET and fMRI co-registration purposes. A sagittal 3D gradient-echo T1-weighted image was acquired using the following sequence: repetition time (TR) = 2.7ms, echo time (TE) = 3.97ms, matrix 256×160, voxel size 1×1×1.5mm^3^. Echo-planar images (EPI) were obtained using a T2*-weighted gradient echo sequence TR = 2.18s, TE = 45ms, 35 axial slices; voxel size 3×3×3mm^3^, flip angle 90°, matrix 64×64). For the fMRI task, stimuli were projected onto a screen at the end of the scanner table, visible through a mirror mounted above the subject’s head. Two magnetic field compatible response boxes were used to record the subject’s responses.

#### Data processing

Functional imaging data were preprocessed and analyzed using Statistical Parametric Mapping (SPM) software (SPM8, Wellcome Trust Neuroimaging Centre, London, UK), implemented in Matlab 7.1.0 (The MathWorks Inc., Natick, MA, USA). Preprocessing included reorientation of the functional images to the anterior commissure, slice time correction, image realignment, co-registration of the T1 scan to the mean image, warping of the co-registered T1 image to Montreal Neurological Institute (MNI) space as defined by SPM’s T1 template, applying the transformations to the slice-timed and realigned images, reslicing to voxels of 3×3×3mm^3^ and applying spatial smoothing using an 8mm full width at half maximum (FWHM) Gaussian kernel. Subject movements of more than 3mm in more than one direction resulted in exclusion of data.

#### Data analysis

In the first level analysis, scanner drifts were modeled using a high pass filter with a cut off of 128s. For each regressor, the onset of the block and the duration of the total block were modeled as a block design, consisting of 22 trial words × [800msec (fixation cross) + 500msec (word presentation) + 900msec (maximum time to press the button)] per word, plus 21 intervals × 32 msec (refresh rate word in scanner), totaling 49.072 ms. Task instructions were modeled separately as a regressor of no interest (Table 1).

The following contrast images were computed:

1) [−2 1 0 1 0 0 0 0] positive classification vs. baseline, in which the positive-neutral (P-O) and neutral-positive (O-P) word pairs were grouped and contrasted to the baseline (italic-regular font pairs and vice versa).
2) [−2 0 1 0 1 0 0 0] negative classification vs. baseline, in which the negative-neutral (N-O) and neutral-negative (O-N) word pairs were grouped and contrasted to the baseline (italic-regular font pairs and vice versa).
3) [−2 0 0 0 0 1 1 0] both emotional valences vs. baseline, in which exclusively emotional valence pairs (P-N and N-P) were grouped and contrasted to the baseline (italic-regular font pairs and vice versa).
4) [−6 1 1 1 1 1 1 0] any emotional valence vs. baseline, in which all emotional valences (P-O, N-O, O-P, O-N, P-N and N-P) were grouped and contrasted to the baseline (italic-regular font pairs and vice versa).

These contrasts were defined for both pre-deprivation and post-deprivation sessions.

Next, on a second level, the contrast images for positive vs. baseline for the pre-deprivation session and the post-deprivation session were entered in a two-sample *t*-test, with session as dependent variable. Additionally, separate models were set up for negative vs. baseline, exclusively emotional valence pairs and any emotional valence vs. baseline. Due to the relative low number of subjects, no additional covariates were entered to these models.

The main effect of time (day 1 vs. day 2) was explored at a threshold of *p* uncorrected <0.005, with an extent threshold of 10 contiguous voxels. Additionally, correction for multiple comparisons was performed by applying Small Volume Correction (SVC) for regions of interest (ROIs) with known involvement in depression, sleep abnormalities and emotional attention. As described in the introduction, the following regions were selected: dorsolateral prefrontal cortex, subgenual cingulate, hippocampal gyrus/ amgydala and insula, defined using the Automated Anatomical Labeling (AAL) system as implemented in the WFU-pickatlas toolbox [27]. Effects occurring in these regions were thus followed up using SVC-correction and results are reported at a Family Wise error (FWE) corrected p-value <.05. Psychometric and performance data (correct responses, false alarms, misses and mean response time for events (RT)) for both days were likewise analysed using paired sample *t*-testing.

### [^11^C]Raclopride PET

#### Data acquisition

[^11^C]Raclopride scans were performed on an ECAT EXACT HR+ scanner (Siemens/CTI, Knoxville, TN, USA). Participants were studied at rest, in supine position, with a nurse nearby and ice cubes in both hands to prevent them from falling asleep. Head movement was restricted by a head immobilization device and Velcro tape. A venous catheter was placed in the forearm for [^11^C]raclopride infusion. A 10min 2D transmission scan using three rotating ^68^Ge/^68^Ga sources was acquired for photon attenuation correction. 370MBq [^11^C]raclopride was dissolved in 5mL saline and administered by an infusion pump (Med-Rad, Beek, The Netherlands), at a rate of 0.8mL·s^−1^, followed by a 35mL saline flush at a rate of 2.0mL·s^−1^. Meanwhile, a 60min dynamic 3D raclopride scan was acquired, consisting of 20 frames with progressively increasing frame lengths (1×15, 3×5, 3×10, 2×30, 3×60, 2×150, 2×300, 4×600s). All PET sinograms were normalized and corrections were applied for decay, dead time, attenuation scatter and randoms. Emission data were reconstructed using FORE+2D filtered back projection [28,29] applying a 5.0mm Hanning filter with a Y-offset of 4cm and a 2.123 zoom. Frames 12–20 were summed (i.e. 5–60min after injection) to create a single frame emission sinogram with high count statistics. Reconstruction of this emission sinogram was performed using ordered-subset expectation maximization (OSEM) with 4 iterations and 16 subsets. OSEM images underwent a 5mm FWHM Gaussian post smoothing, to obtain a transaxial spatial resolution of 7mm FWHM, equal to that of filtered back projected (FBP) images. Final images consisted of 63 planes of 128×128 voxels, each 2.4×2.4×2.4 mm^3^.

#### Data processing

All structural MRI scans were rotated to the axial (horizontal) plane, parallel to the anterior and posterior commissure (AC–PC) line. To correct for possible motion, each frame (1–20) was coregistered to the summed image over frames 12–20. These motion corrected PET images were subsequently coregistered to the realigned MRI scan using Volume Imaging in Neurological Research (VINCI) software [30].

#### Kinetic analysis

Mean non-displaceable binding potential (BP_ND_) was used as a measure of dopamine D2/D3 receptor availability. Using the in-house developed software package PPET [31], parametric BP_ND_ images were generated using receptor parametric imaging (RPM2), a basis function implementation of the simplified reference tissue model (SRTM) [32]. Cerebellum grey matter was used as reference tissue, for which automated cerebellar volumes of interest (VOIs) were defined using partial volume effect (PVE) lab [33]. This analysis also provided parametric R_1_ images, representing local tracer delivery relative to that to the cerebellar reference region. Basis function settings used were: start exponential = 0.05min^−1^, end = 0.5min^−1^, number of basis functions 32.

#### Statistical parametric mapping

Parametric BP_ND_ images were analyzed using SPM8. After spatial preprocessing, including reorientation and normalization to MNI space, images were analyzed on a voxel by voxel basis, using a basal ganglia mask created with WFU Pickatlas software [27]. No proportional scaling was applied. SPM RPM2 and R_1_ BP_ND_ images were entered in paired sample *t*-tests. The threshold was set at *p* uncorrected ≤0.005 with an extent threshold of 10 voxels.

## Results

### Psychometric Data

At baseline, depressive symptoms were low to absent (BDI score 1.8 ± 2.0). Using the Spielberger State-Trait Inventory (STAI), containing 20 items to be scored on a four-point Likert scale (range 20–80), mean trait anxiety score was 29.4 ± 4.8 and state anxiety at baseline scanning 30.4 ± 3.9. During the TSD night, VAS energy levels declined significantly (F(1,11) 20.2, *p* = 0.001), in line with decreased concentration (F(1,11) 10.6, *p* = 0.01), speed of thought (F(1,11) 12.0, *p* = 0.007), and increased perceived motor retardation (F(1,11) 12.0, *p* = 0.007), but not significantly for mood (F(1,11) 2.9, *p* = 0.122). STAI scores indicated a trendwise increased anxiousness after TSD, 36.3 ± 10.7 (*p* = 0.068).

### Cortisol Data

**Figure 1:**
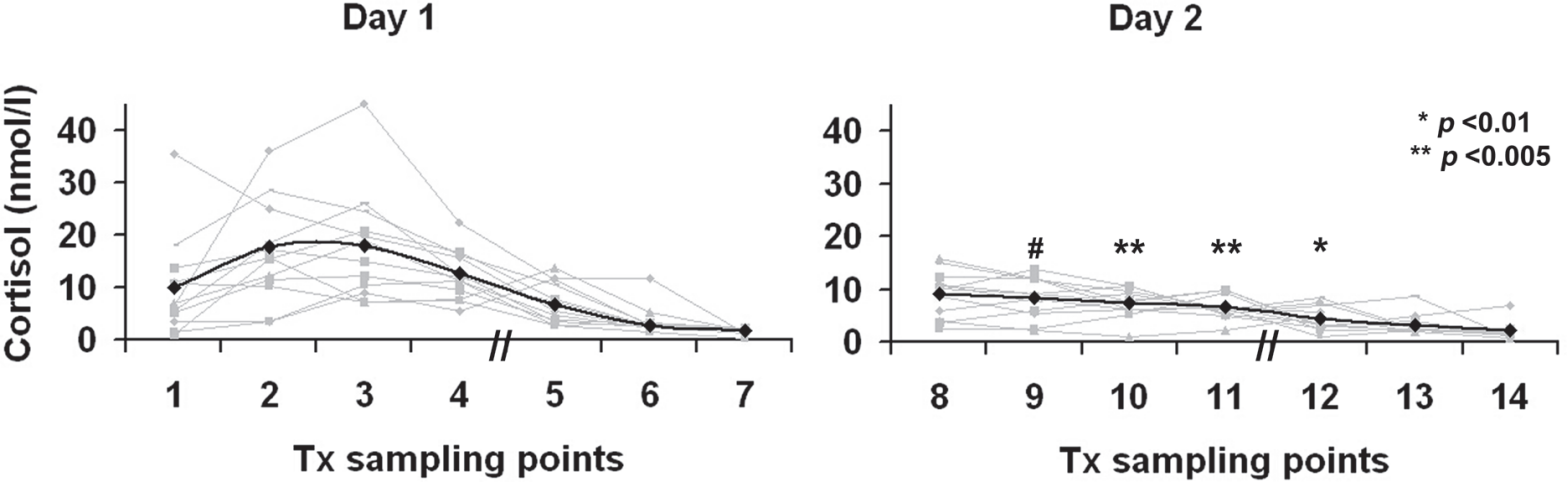
Effects of total sleep deprivation on saliva cortisol levels. Individual saliva cortisol curves (grey line) and cortisol mean value (nmol/L) per Tx sampling point (solid line). Day 1 shows baseline cortisol sampling at T1-T7, day 2 shows effects of one night of total sleep deprivation on cortisol levels at T8-T14. T1, 2 and 3 comprise the cortisol awakening response (CAR). T8, 9 and 10 are sampled at identical time points the following day. T5 and T12 are sampled at 14.00hr, T6 and T13 at 17.00hr and T7 and T14 at 23.00hr. *p* values show effects of TSD, # *p* = 0.016.

After TSD, CAR AUC_I_ and AUC_G_ showed significant blunting (*p* = 0.029 and *p* = 0.022, respectively) (Table 2, Fig. 1). On day 1, nine subjects showed a rise in cortisol during the first hour after awakening, compared with a much smaller increase in five subjects after TSD, signified by a decreasing MnInc CAR. Similarly, cortisol AUC_G_ T1-7 vs. T8-14 showed a robust decline after TSD. Cortisol levels were normally distributed on both days and showed no significant gender differences. Evening cortisol was not discriminating.

**Table 2:** Saliva cortisol summary indicators.

**Day**	**Indicator**	**Mean ± Sd**	**Min**	**Max**	**Day**	**Indicator**	**Mean ± Sd**	**Min**	**Max**	***p***
1	CAR AUC_I_	292.08 ± 443.44	−520.35	1290.15	2	CAR AUC_I_	−40.65 ±105.65	−170.25	126.00	0.029
	CAR AUC_I_/ hr	4.87 ± 7.39	−8.67	21.50		CAR AUC_I_/ hr	−0.68 ± 1.76	−2.84	2.10	
	CAR AUC_G_	886.58 ± 472.21	233.10	1677.15		CAR AUC_G_	511.50 ± 208.95	127.95	798.45	0.022
	CAR AUC_G_/ hr	14.78 ± 7.87	3.89	27.98		CAR AUC_G_/ hr	8.53 ± 3.48	2.13	13.31	
	MnInc CAR	6.72 ± 10.51	−12.16	30.30		MnInc CAR	−1.05 ± 2.48	−4.26	3.16	0.030
	AUC_G_ T1–7	6286 ± 2129	2824	11026		AUC_G_ T8–14	4323 ± 783	2857	6085	0.003
	AUC_G_ T1–7 / hr	6.55 ± 2.22	2.94	11.49		AUC_G_ T8–14 / hr	4.50 ± 0.82	2.98	6.34	

*n* = 162/168 (94.5%) cortisol saliva samples. Units are nmol/l. *p* = paired samples *t*-test for cortisol summary indicators day 1 versus day 2.

AUC_I_, area under the curve, relative to increase, units nmol/l/min, or nmol/l/hr; AUC_G_, area under the curve, relative to zero, units nmol/l/min, or nmol/l/hr; CAR, cortisol awakening response, including samples at awakening (T1), +30 (T2) and +60 (T3) minutes after awakening; MnInc, Mean Increase in 1^st^ hour after awakening = ((sample T2 + T3)/(2)) – T1.

### fMRI

Twelve data sets were available on day 1, and 11 on day 2 due to scanner logistic problems. After TSD, subjects were significantly slower in reacting during the neutral condition (*p* = 0.043), but also to positive targets with a neutral distracter (*p* = 0.008). The proportion of correct versus false answers decreased trendwise for neutral words (*p* = 0.082) and for positive targets with a negative distracter (*p* = 0.079) (Table 3). *Post hoc*, results were additionally analyzed using general linear model statistics (GLM). When performing multivariate testing, the effect of sleep deprivation on reaction time for emotional words was significant at F(1,20) = 34.14, *p*<0.001; the effect of time for sleep deprivation was significant at F(1,20) = 5.78, *p* = 0.037, indicating that participants were slower at day 2, due to sleep deprivation. The interaction effect of sleep deprivation on emotion* time was not significant (F(1,20) = 0.81, *p* = 0.475), indicating that the general slowing following deprivation was common for all emotions presented.

**Table 3:** fMRI task results.

**Condition**		**Reaction time (msec) correct answers**			**Proportion correct-false**		
**Target**	**Distracter**	**Day 1**	**Day 2**		**Day 1**	**Day 2**	
		**Mean ± SD**	**Mean ± SD**	***p* value**	**Mean ± SD**	**Mean ± SD**	***p* value**
Italics (I) Regular (R)	Regular (R) Italics (I)	460 ± 98	504 ± 96	0.043*	0.93 ± 0.05	0.87 ± 0.10	0.082†
Positive (P)	Neutral (O)	635 ± 144	713 ± 174	0.008**	0.77 ± 0.12	0.75 ± 0.17	0.609
Negative (N)	Neutral (O)	695 ± 172	701 ± 164	0.783	0.80 ± 0.13	0.76 ± 0.17	0.606
Neutral (O)	Positive (P)	713 ± 208	754 ± 181	0.449	0.84 ± 0.15	0.80 ± 0.13	0.578
Neutral (O)	Negative (N)	730 ± 176	760 ± 176	0.215	0.77 ± 0.23	0.68 ± 0.26	0.306
Positive (P)	Negative (N)	665 ± 198	686 ± 149	0.541	0.92 ± 0.06	0.87 ± 0.09	0.079†
Negative (N)	Positive (P)	673 ± 173	674 ± 134	0.977	0.91 ± 0.07	0.88 ± 0.10	0.376

*n* = 11 pairs

*p* = paired sample *t*-test, two-sided

† = *p*<0.10

* = *p*<0.05

** = *p*<0.01

**Figure 2:**
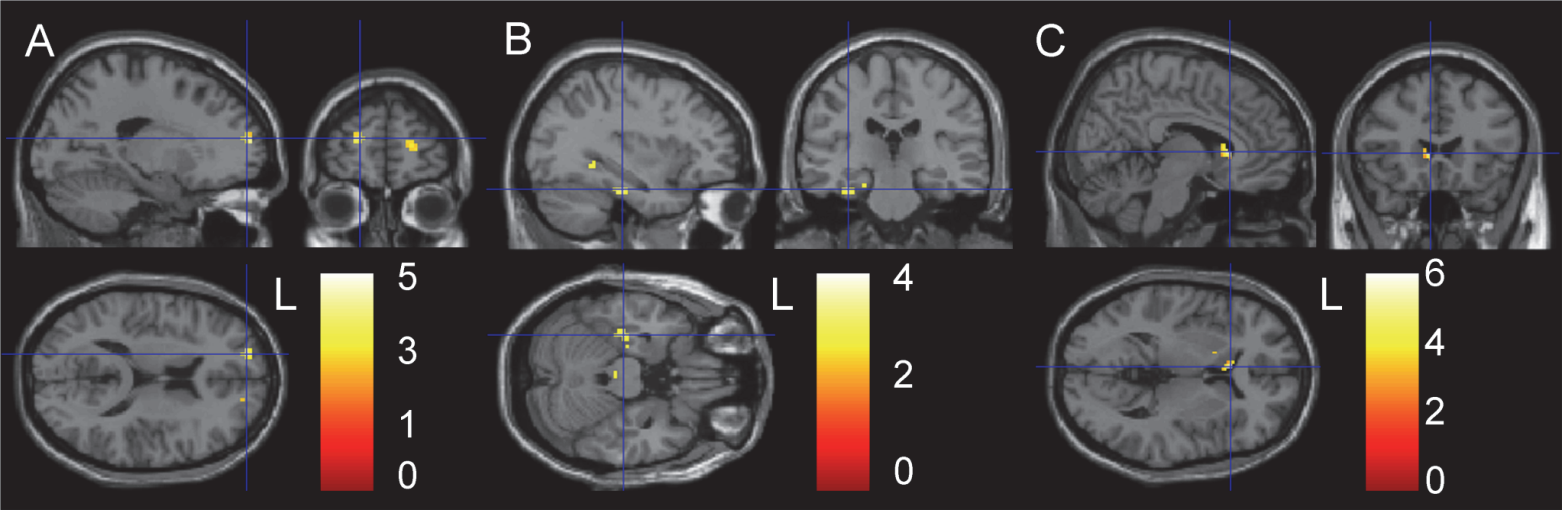
Effects of TSD for fMRI and [^11^C]raclopride PET. *p* <0.005, extent threshold 10 voxels. A and B are task related fMRI results, showing increased prefrontal and limbic activation respectively, in the conditions (A) positive valence versus baseline and (B) both emotional valences. C is a [^11^C]raclopride PET image, showing decreased voxel-by-voxel RPM2 binding potential (BP_ND_) in nucleus caudatus in *n* = 8. At the bottom right is the Z-score scale depicted.

After 25 hours of wakefulness, the neutral condition showed no significant activation differences at a group level (Table 4). Evaluation and processing of positive words was associated with increased bilateral prefrontal activation in addition to increased activation of left medial prefrontal working memory areas (Fig. 2A). Left DLPFC activation remained significant after Small Volume Correction (SVC; AAL *p*_FWE_ 0.02). Processing of negative words was associated with increased activity in left insular area, but this effect did not survive SVC. During conditions containing emotional words only, viz. positive targets and negative distracters (P-N), or vice versa (N-P), left insular, limbic and parahippocampal lobes were activated, as well as right parietal lobe (Fig. 2B), showing SVC subthreshold increased activation in the hippocampal/parahippocampal region. All emotional conditions (i.e. target and/or distracter) resulted in increased activation in the anterior part of the left insula (AAL *p*_FWE_ 0.043), mainly driven by the response to words with a negative valence, in addition to activation of the parietal lobe.

**Table 4:** Neuroimaging effects of TSD for fMRI and PET.

	**K_E_**	**Z-score**	***p* uncorr**	**AAL Region**	**L/R**	**Region**	**x**	**y**	**z**
				***p*_FWE_ corr**					
**Effects for fMRI, TSD >baseline**									
P-O and O-P	15	4.03	0.000	0.020	L	Superior frontal gyrus	−18	57	15
	10	3.36	0.000	0.202	L	Middle frontal gyrus	−30	42	21
	11	3.35	0.000		L	Medial frontal gyrus	−12	9	45
	12	3.04	0.001	0.363	R	Superior frontal gyrus	21	54	9
N-O and O-N	10	3.71	0.000	0.176	L	Insula	−42	0	15
	14	3.05	0.001	0.449	R	Superior frontal gyrus	15	57	12
P-N and N-P	11	3.66	0.000	-	R	Parietal cortex	21	−42	39
	36	3.55	0.000	0.142	L	Hippocampal gyrus	−24	−36	−6
	19	3.41	0.000	0.051	L	Parahippocampal gyrus	−33	−24	−24
				0.089	L	Parahipp + Hippocampal gyrus	−33	−24	−24
	11	3.18	0.001	-	L	Anterior insula	−27	−9	18
				-	L	Amygdala			
P-O, O-P, N-O, O-N, P-N, N-P	17	3.62	0.000	0.043	L	Anterior insula	−39	−3	12
	13	3.54	0.000	-	R	Parietal cortex	21	−42	42
I-R and R-I	-	-	-		-	-	-	-	-
**[^11^C]Raclopride PET, TSD <baseline (SPM)**									
RPM2 BP_ND_	56	3.82	0.000		L	AAL-Nucleus caudatus	−8	22	4
R_1_	88	3.93	0.000		R	AAL-Nucleus caudatus	14	0	20

*p*<0.005, extent threshold 10 voxels. P is a positive valence word; O is a neutral valence word; N is a negative valence word; I is a neutral valence word, written in italics; R is a neutral valence word, written in regular script. Any valence is either target or distracter.; Small Volume Correction, using anatomical automatic labeling (AAL) as in WFU Pickatlas; BP_ND_, Binding potential; K_E_, number of voxels in cluster; L, left; R, right; R_1_, relative delivery; RPM, receptor parametric mapping; TSD, total sleep deprivation.

### [^11^C]Raclopride

A subset of 8 paired data sets was available due to a failed synthesis (1 TSD scan) and technical problems with 1 baseline and 2 TSD scans. For *n* = 8, injected masses of raclopride were 2.36 ± 1.08 and 1.45 ± 0.55μg, on days 1 and 2 respectively (*p* = 0.06) and injected doses of [^11^C]raclopride were 378 ± 12 and 390 ± 19MBq on days 1 and 2, respectively (*p* = 0.230). TSD resulted in a significantly decreased voxel-by-voxel based BP_ND_ in left caudate nucleus, as shown in Table 4 and Fig. 2C. In addition, there was a TSD induced decrease in R_1_ in right caudate nucleus.

### Clinical Interactions

*Post hoc* we tested for correlations for TSD related changes in cortisol AUC, regions of interest (ROI) based BOLD response to emotional words, and altered [^11^C]raclopride binding, using anatomical automatic labeling (AAL) defined striatal regions, according to WFU Pick atlas [27]. No statistically significant correlations were observed.

## Discussion

In the present study the effects of total sleep deprivation on stress regulating brain systems in healthy subjects were investigated as preliminary work for a TSD study in mood disorder. During a sleep deprived night, VAS scores on energy, concentration and speed of thought, but not mood, declined significantly. Although at baseline participants were not clinically depressed or overly anxious, as witnessed by BDI and STAI-scores, validated instruments like the Positive and Negative Affect Schedule (PANAS)[34,35] and the Profile of Mood State (POMS)[36] could have been used to score a broad range of mood states, both at baseline and during the night of sleep deprivation and the subsequent day.

After 24 hours of prolonged wakefulness, significant blunting of the cortisol awakening response (CAR) and secretion over the day (AUC_G_) were found. Normally, under the influence of the SCN, HPA activity increases during the night, resulting in a cortisol rise two to three hours after sleep onset, which continues to rise into the early waking hours [12,14]. The present results indicate robust attenuation of the HPA-mediated stress response after TSD, congruous with decreasing VAS scores and lowered arousal, which may be due to the absence of the initial physiological awakening response [12,37]. These findings are in line with Vzontgas and colleagues, finding lowered, albeit not significantly, 24 hour plasma cortisol levels in blood in a laboratory setting in a group of 10 men [38].

In the present study, no significantly altered cortisol levels were found after 14.00h (T5-T12). Evening cortisol, indicating return to baseline levels, was slightly lower than those reported by Vreeburg [19] in healthy subjects.

In order to investigate effects of TSD on processing of both positive and negative stimuli, as well as on cognitive inhibition, we chose to adapt the Murphy and Elliott fMRI paradigm [24,25,39,40], as this task was originally developed to investigate emotional bias in mood disorders in the context of cognitive processing. After TSD, in healthy adults, task performance during fMRI was slower, indicating that TSD overruled any learning or practice effects. Slowing of task performance after TSD is in line with previous reports and likely due to loss of sustained attention and vigilance [41]. Slowing was particularly evident for positive targets with a neutral distracter. Accuracy was trendwise decreased for the neutral (italic vs. regular font) condition and for positive targets with negative distracters, suggesting decreased sensitivity to detect positive valence. On processing emotionally salient versus neutral words, TSD was associated with increased left dorsolateral prefrontal activity, suggesting increased mental effort to perform semantic judgements and to maintain control, in a setting of less efficient functional circuitry. Although we do not intend to overstate the relevance of these findings in this emotionally healthy group, cognitive biases in depressive disorder are thought to reflect maladaptive bottom-up processes, which are generally perpetuated by weakened cognitive control [42]. Processing of solely affective stimuli (target and distracter) showed subthreshold increased activation in the left parahippocampal /hippocampal region. Activation of the subgenual gyrus and amygdala was remarkably absent, though for amygdala this is line with findings by Elliott et al., [25], fostering [presumably reflecting] a lower affective salience for words compared to pictures. Processing of any affective stimulus (target and/or distracter) showed increased activation of the anterior part of the insula in a context of performance anxiety as indicated by trendwise increased STAI scores in these healthy, but weary adults [43]. Activation of the insula was mainly driven by the response to words with a negative valence, suggestive of an increased effort to handle negative affect [1] and in line with the insular function of emotional interference resolution in working memory [44]. With due caution, we propose that these neural responses reflect modulation of cognitive performance by emotional tone. Therefore, these regions likely represent an interface between cognition and emotion processing [25].

After TSD, voxel-by-voxel based BP_ND_ of [^11^C]raclopride was significantly decreased in left caudate, which is partly in accordance with a report by Volkow [9]. This was not explained by regional altered delivery (R_1_) of the tracer, although metabolic activity in the cerebellar reference tissue may be altered after sleep deprivation [6,7]. A reduction in [^11^C]raclopride specific binding is consistent with either an increase in dopamine release, or a decreased affinity of the synaptic D2/D3 receptor in these regions [45], which may be due to internalization of receptors [46]. This could not be determined on the basis of our design, and may have resulted from a combination of these factors.

Using both [^11^C]raclopride and a dopamine transporter blocking radioligand, [^11^C]methylphenidate, Volkow [10] argued TSD induced decreased [^11^C]raclopride binding not to be due to increased dopamine availability, but to decreased affinity of the D2/D3 receptor, resulting in dopamine receptor downregulation in the synaptic cleft. As dopamine D2 receptors are thought to be involved in wakefulness, and partially responsible for maintaining arousal and alertness [8,47], the present reduced VAS on energy and concentration and efficiency in fMRI task performance, are in line with D2 down-regulation. This would further be exemplified by the blunted cortisol response, since dopaminergic stimulation of the HPA axis is mediated through D1 and D2 receptors [11]. Decreased affinity in the head of the left caudate could be in line with increased difficulty in controlling word interference from task unrelated processing [48], explaining both the general slowing and increased prefrontal activity. However, we were not able to corroborate this explanation in a correlational analysis, which may be primarily due to insufficient power, but may also indicate that regional brain activation as measured with fMRI is not tightly coupled to either striatal dopaminergic transmission or HPA axis activity. Excluding two smokers did not change results significantly, although smoking may influence dopamine release and therefore raclopride binding [49].

### Clinical Relevance

Individual vulnerability to sleep deprivation is known to be variable [3]. From the present study, it cannot be ruled out that decreased D2 receptor affinity is the brain’s response to initially increased dopamine levels, induced by TSD. Blunting of the HPA axis response may reflect the absence of awakening stress and possibly explain some of the beneficial effects of sleep deprivation in depressive mood disorder.

### Limitations

This pilot study in healthy adults contains several potential limitations. In view of our modest sample size and fixed-order design, the current results are in clear need of replication.

Regarding baseline characteristics, the participants’ number of hours of sleep was adequate at the start of the experiment, but we did not control objectively for sleep quality and duration. Baseline CAR may have been affected by waking up earlier, or by the excitement of taking part in a research study, which may have released additional ACTH [50]. A higher CAR has been associated with shorter sleep duration [51]. However, excluding three subjects who slept 6 hours or less, did not have a major effect on the CAR (*p* = 0.022). During the night, participants were kept in a well-lit room. Melatonin suppression may have dampened the SCN-mediated CRH response.

For our fMRI runs, we have chosen to adapt the original Murphy and Elliott task [24,25], who described their paradigm to investigate emotional bias in depressive disorder as a go/no-go task. However, go/no-go paradigms do not typically feature an even split of valid and invalid targets, and therefore we have renamed the task as a semantic affective classification task. The task was modeled as a block design, and because the inter-stimulus interval (ISI) was fixed, could not be analyzed as an event-related design. Evidently, a block design is preferable when sample sizes are modest, as it is generally more robust [52], although it lacks the flexibility of event-related designs. Therefore, for assessing individual cognitive and emotional responses in e.g. a patient population, an event-related design would be more appropriate [53]. Finally, for our voxel-based analyses we set an *a priori* threshold of *p* = 0.005 and 10 voxels to obtain a reasonable balance between Type I and Type II error [54], again highlighting the need for a replication in a larger sample.

With respect to mood enhancers, other drugs of abuse were not tested for. At baseline, we did not control for caffeine use at home before the start of the experiments. Caffeine evokes its stimulating effects through blockade of the adenosine receptor [55], which in turn is involved in the control of dopamine release [56]. As raclopride is a dopamine receptor antagonist, in theory, TSD induced changes in raclopride binding may therefore have been underestimated.

Although changes in [^11^C]raclopride BP_ND_ clearly show a dose dependent relationship with extracellular DA levels, the nature of this relationship is complex [57]. [^11^C]Raclopride BP_ND_ does not differentiate between binding to receptors in high or low affinity states, whereas endogenous dopamine is mainly conveyed by high affinity state receptors [58], acting on pre- and postsynaptic (extra)-striatal dopaminergic D1 receptors to bring about its effect [59]. Therefore, dopaminergic effects due to TSD may have been underestimated and future research should resolve this issue, for example by comparing [^11^C]raclopride to the purported high-affinity ligand [^11^C]PHNO [60]. As [^11^C]raclopride scans were performed in the second half of the morning, and the time sequence of dopamine release is not known, effects may have been either over- or underestimated. A variable response to TSD is in line with observations in depressed patients, where the therapeutic response to TSD may vanish within hours to a day [3].

## Conclusion

Sleep deprivation in healthy adults induces widespread neurophysiological and endocrine changes, characterized by impaired cognitive functioning, despite increased regional brain activity. Our pilot findings indicate that activation of the dopaminergic system occurs together with a blunted cortisol response, suggesting augmented motivational top down control and requiring increased involvement of prefrontal and limbic cortical areas. Sustained wakefulness requires the involvement of compensatory brain systems, and may help to understand the therapeutic effects of sleep deprivation in affective disorders.
